# Supplementary material for: Triple stable isotope analysis to estimate the diet of the Velvet Scoter (Melanitta fusca) in the Baltic Sea
Source: PeerJ. 2018 Jun 27;6:e5128. doi: 10.7717/peerj.5128 (PMC6026463; doi:10.7717/peerj.5128)
Supplement: Appendix S1 [file peerj-06-5128-s002.docx]

Trophic enrichment factors applied for the Model0.

| Source | Mean ∆δ^34^S | SD ∆δ^34^S | Mean ∆δ^13^C | S SD ∆δ^13^C | Mean ∆δ^15^N | SD ∆δ^15^N |
| --- | --- | --- | --- | --- | --- | --- |
| *Saduria entomon* | 0.00 | 0.00 | -0.24 | 0.06 | 2.25 | 0.20 |
| *Crangon crangon* | 0.00 | 0.00 | 0.01 | 0.02 | 2.25 | 0.20 |
| *Mya arenaria & Cerastoderma glaucum* | 0.00 | 0.00 | 0.29 | 0.04 | 2.25 | 0.20 |
| *Macoma balthica* | 0.00 | 0.00 | 0.38 | 0.12 | 2.25 | 0.20 |
| *Polychaetes* | 0.00 | 0.00 | 0.43 | 0.10 | 2.25 | 0.20 |
