# Supplementary figures and images for: Triple stable isotope analysis to estimate the diet of the Velvet Scoter (Melanitta fusca) in the Baltic Sea
Source: PeerJ. 2018 Jun 27;6:e5128. doi: 10.7717/peerj.5128 (PMC6026463; doi:10.7717/peerj.5128)

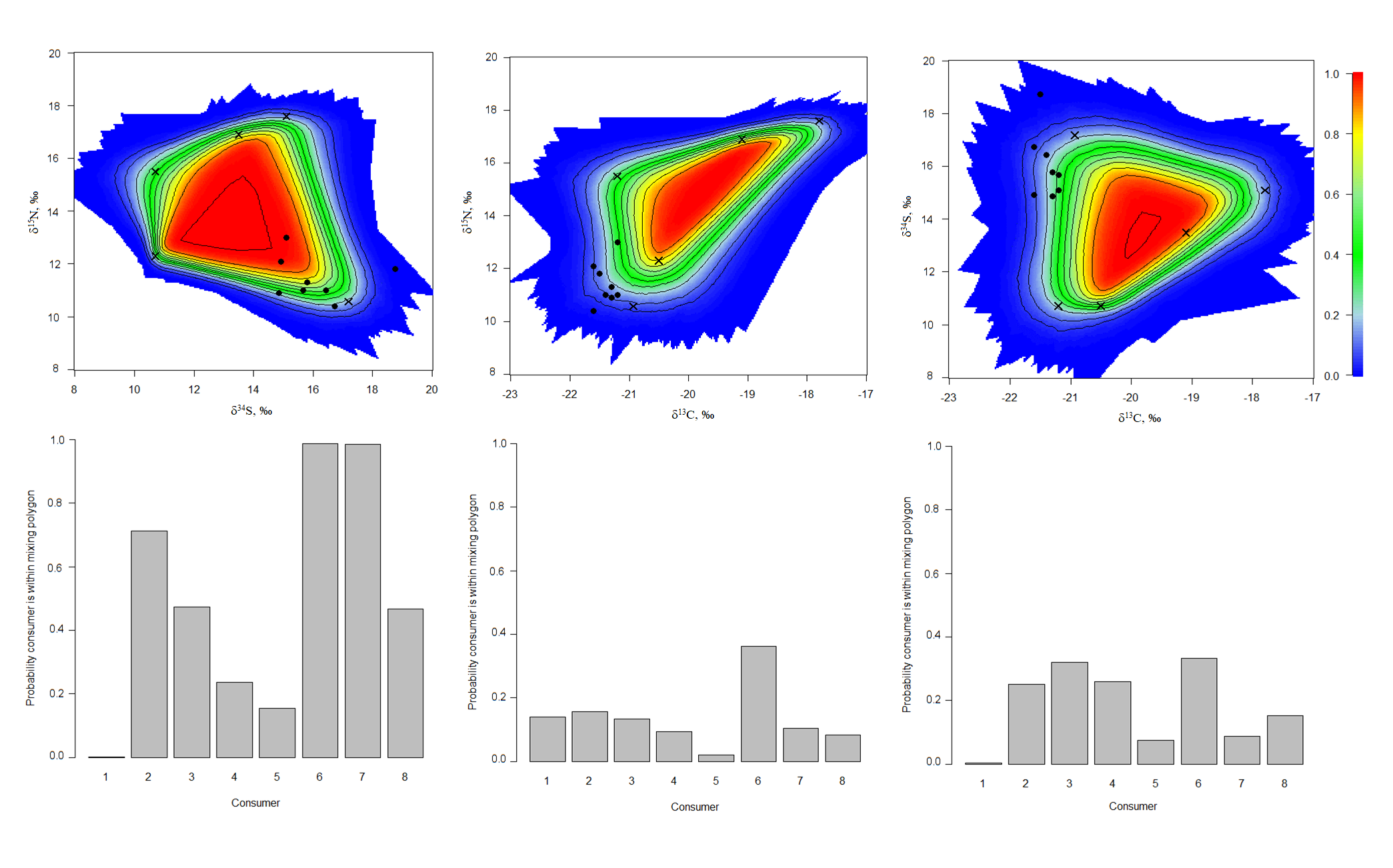

Supplement: Appendix S2 [file peerj-06-5128-s003.png]

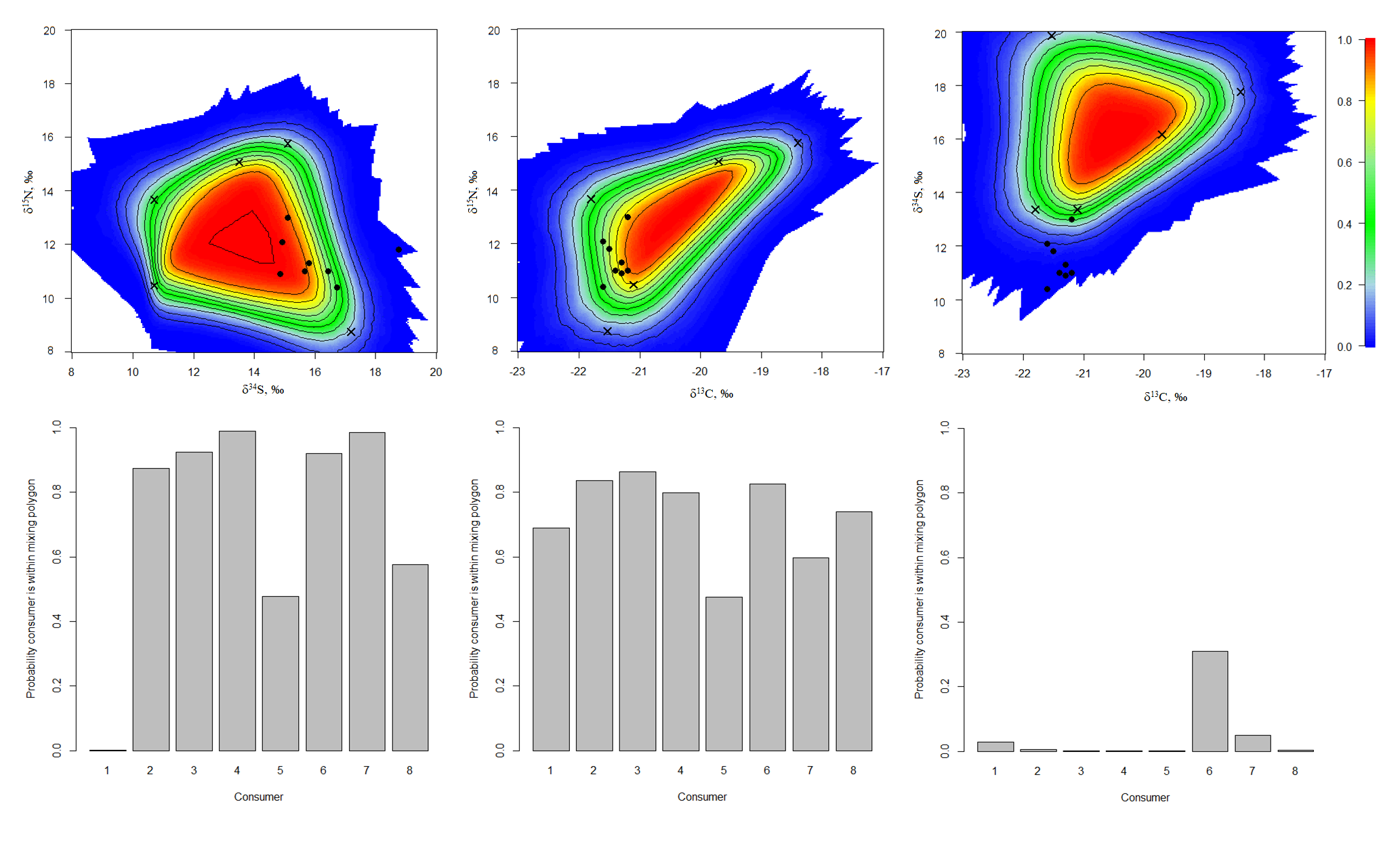

Supplement: Appendix S3 [file peerj-06-5128-s004.png]

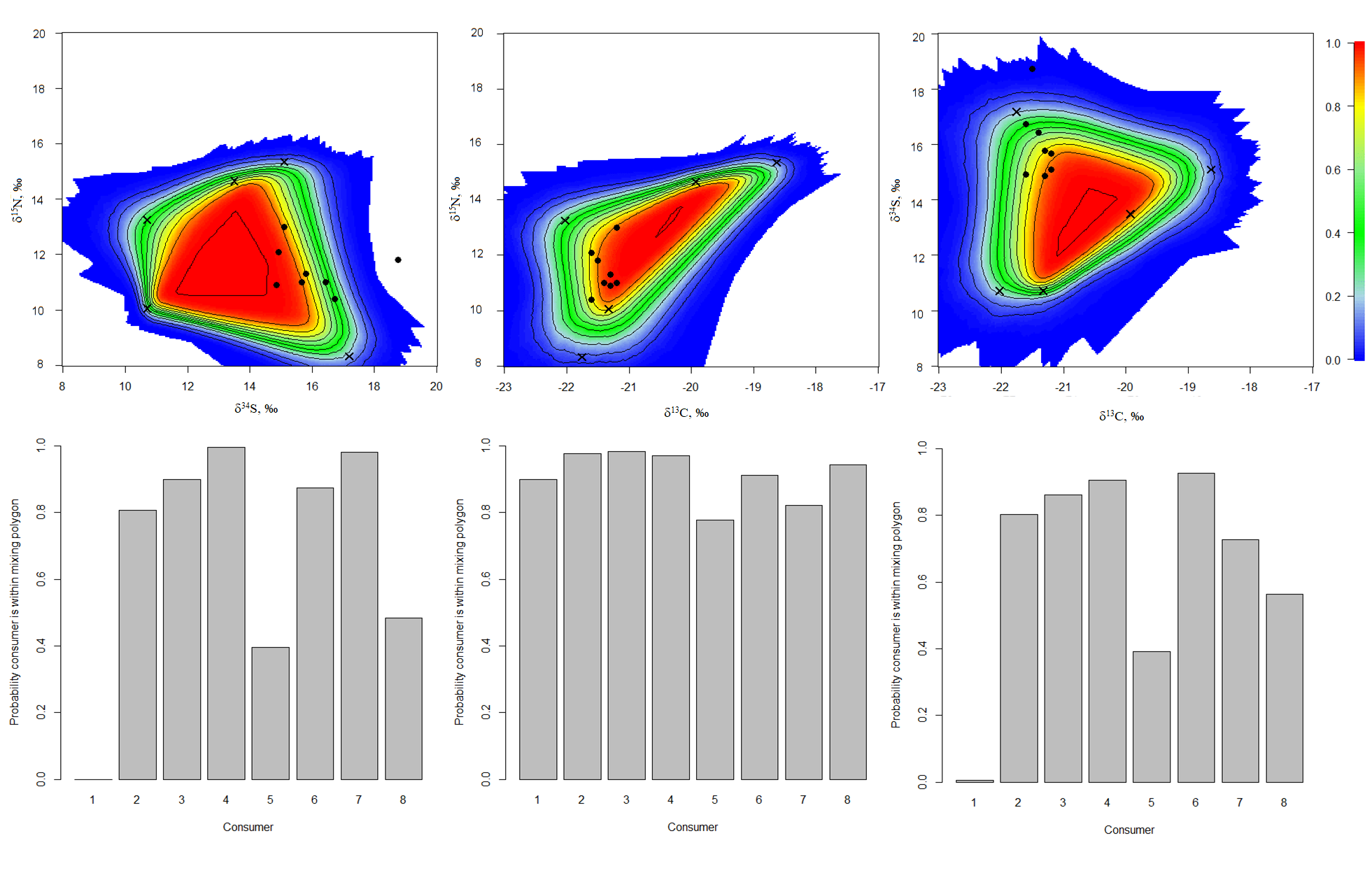

Supplement: Appendix S4 [file peerj-06-5128-s005.png]
