# Supplementary material for: Triple stable isotope analysis to estimate the diet of the Velvet Scoter (Melanitta fusca) in the Baltic Sea
Source: PeerJ. 2018 Jun 27;6:e5128. doi: 10.7717/peerj.5128 (PMC6026463; doi:10.7717/peerj.5128)
Supplement: Appendix S5 — Different sets of prior information as the wet weight (WW) and the organic matter weight (AFDW) of food objects from gut contents analysis. [file peerj-06-5128-s006.docx]

Contributions of food sources to the diet of velvet scoters, calculated by five source mixing model (ModelC) using triple stable isotope values of δ^34^S, δ^13^C and δ^15^N and different sets of prior information as wet weight (WW) or organic matter weight (AFDW) of food objects from gut contents analysis.

| Sources | Proportions, % as Mean±SD (CI_95_) | | |  |
| --- | --- | --- | --- | --- |
|  | No prior information | WW | AFDW |  |
| *Saduria entomon* | 9 ± 6 (0-20)* | 35 ± 4 (27-43) | 24 ± 5 (16-34)* |  |
| *Crangon crangon* | 15 ± 9 (0-31)* | 0,3 ± 0,5 (0-1) | 1 ± 1 (0-4) |  |
| *Mya arenaria & Cerastoderma glaucum* | 51 ± 9 (32-67)* | 46 ± 4 (38-53) | 56 ± 5 (46-65)* |  |
| *Macoma balthica* | 7 ± 7 (0-21) | 16 ± 3 (9-22) | 16 ± 3 (10-23) |  |
| *Polychaetes* | 17 ± 9 (0-33)* | 3 ± 2 (0-6) | 2 ± 2 (0-6) |  |

* shows negligible difference between outputs of ModelC (this table) and Model0 (Table 5 in the main text).
